# (3*R*,4*S*)-3-Methyl-4-phenyl-2-[(*R*)-1-phenyl­eth­yl]-3,4-di­hydro­isoquinolin-2-ium tetra­fluorido­borate

**DOI:** 10.1107/S160053681400230X

**Published:** 2014-02-08

**Authors:** Karim Ben Ali, Pascal Retailleau

**Affiliations:** a Académie Militaire, Fondouk Jedid, 8012 Nabeul, Tunisia; b Centre de Recherche de Gif sur Yvette, ICSN-CNRS, 1 avenue de la Terrasse, 91198 Gif sur Yvette, France

## Abstract

The title salt, C_24_H_24_N^+^·BF_4_^−^, is one of two possible dias­tereoisomers having a different configuration of the asymmetric centre in the α-phenyl­ethyl substituent, whose absolute configuration was established to be *R*. The two phenyl substituents of the cation have a cofacial orientation, albeit with a long centroid–centroid separation of 4.129 (3) Å. The crystal structure exhibits numerous C—H⋯F contacts between counter-ions, with the tetra­fluorido­borate anion surrounded by five iminium cations.

## Related literature   

For related literature, see: Adam *et al.* (2001 ▶); Bohé *et al.* (1999 ▶); Cremer & Pople (1975 ▶); Davies & Coote (1988 ▶)
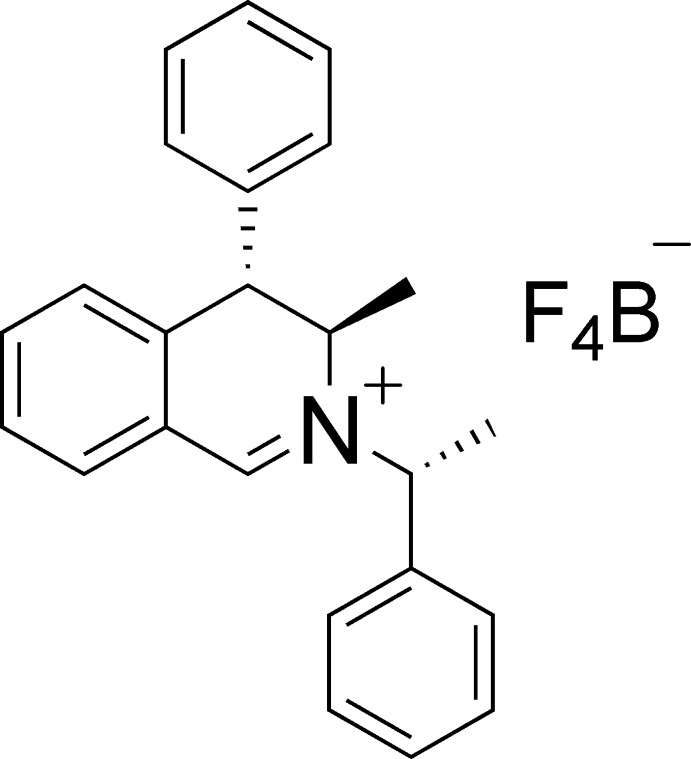


## Experimental   

### 

#### Crystal data  

C_24_H_24_N^+^·BF_4_^−^
*M* *_r_* = 413.25
Tetragonal, 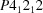

*a* = 9.367 (4) Å
*c* = 49.137 (14) Å
*V* = 4311 (3) Å^3^
*Z* = 8
Cu *K*α radiation
μ = 0.81 mm^−1^
*T* = 293 K
0.33 × 0.26 × 0.26 mm

#### Data collection  

Enraf–Nonius CAD-4 diffractometer
10992 measured reflections
2353 independent reflections
1192 reflections with *I* > 2σ(*I*)
*R*_int_ = 0.092
3 standard reflections every 60 min intensity decay: 1%

#### Refinement  

*R*[*F* ^2^ > 2σ(*F* ^2^)] = 0.065
*wR*(*F* ^2^) = 0.215
*S* = 1.08
2353 reflections
273 parameters
H-atom parameters constrained
Δρ_max_ = 0.30 e Å^−3^
Δρ_min_ = −0.18 e Å^−3^

### 

Data collection: *CAD-4 Software* (Enraf–Nonius, 1989 ▶); cell refinement: *CAD-4 Software*; data reduction: *NONIUS* (Riche, 1989 ▶); program(s) used to solve structure: *SHELXS97* (Sheldrick, 2008 ▶); program(s) used to refine structure: *SHELXL97* (Sheldrick, 2008 ▶); molecular graphics: *ORTEP* (Johnson, 1965 ▶), *PLATON* (Spek, 2009 ▶) and *Mercury* (Macrae *et al.*, 2008 ▶); software used to prepare material for publication: *publCIF* (Westrip, 2010 ▶).

## Supplementary Material

Crystal structure: contains datablock(s) I, global. DOI: 10.1107/S160053681400230X/ld2119sup1.cif

Structure factors: contains datablock(s) I. DOI: 10.1107/S160053681400230X/ld2119Isup2.hkl

CCDC reference:

Additional supporting information:  crystallographic information; 3D view; checkCIF report

## Figures and Tables

**Table 1:** Hydrogen-bond geometry (Å, °)

*D*—H⋯*A*	*D*—H	H⋯*A*	*D*⋯*A*	*D*—H⋯*A*
C1—H1⋯F27	0.93	2.39	3.292 (9)	164
C1—H1⋯F30	0.93	2.55	3.170 (12)	125
C18—H18⋯F29	0.98	2.55	3.292 (10)	132
C24—H24⋯F30^i^	0.93	2.60	3.430 (15)	148
C4—H4⋯F27^ii^	0.98	2.54	3.498 (8)	165
C11—H11*A*⋯F28^ii^	0.96	2.50	3.268 (11)	137
C14—H14⋯F28^iii^	0.93	2.54	3.249 (10)	133
C7—H7⋯F27^iv^	0.93	2.65	3.399 (10)	138

Symmetry codes: (i) 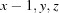; (ii) 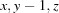; (iii) 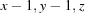; (iv) 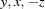.

**Fig. 1.**
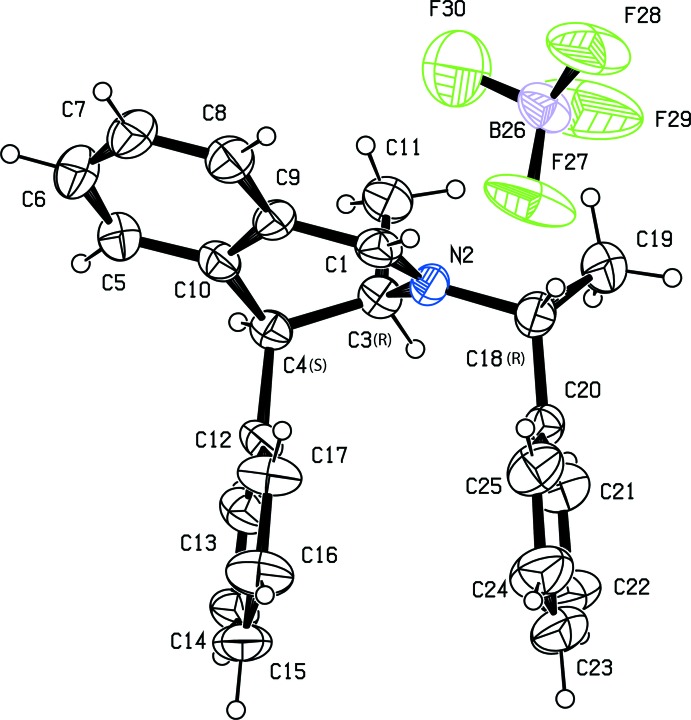
An ORTEP view of the title compound showing the atom-labelling scheme. Displacement ellipsoids are drawn at the 30% probability level. H atoms are represented as small spheres of arbitrary radii.

**Fig. 2.**
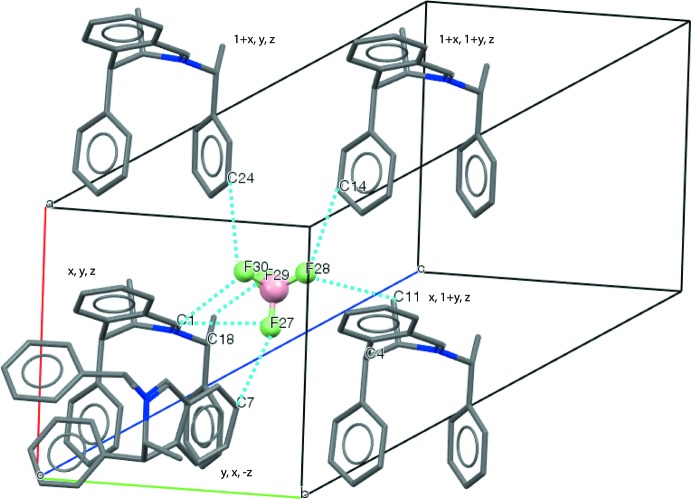
Environment of the BF4- anion in the crystal of the title compound. Geometric parameters for the non-conventional C–H···F hydrogen bonds shown as cyan dotted lines are provided in supplementary material section.

## supplementary crystallographic information

### 1. Comment

In recent years, much effort has been devoted to the development of organocatalytic processes that afford metal-free procedures (Adam *et al.*, 2001), such as asymmetric epoxidation catalysed by chiral iminium salts. It has been proved that iminium salts are effective catalysts at low loadings for enantioselective epoxidation using Oxone® (2KHSO_5_.KHSO_4_.K_2_SO_4_) as the stoichiometric oxidant.

As a part of our interest in catalytic epoxidation by dihydroisoquinolinium-derived iminium salts (Bohé *et al.*, 1999), we report herein the synthesis from the corresponding (3R,4S)-3-methyl-4-phenyl-3,4-dihydroisoquinoleine (Davies *et al.*, 1988), by N-alkylation with (1-bromoethyl)benzene, and the crystal structure determination of the title compound. It was isolated as one of the two diastereoisomeric products; it is a new dihydroisoquinolinium-derived iminium salt containing an asymmetric centre in an exocyclic substituent at the nitrogen atom. The X-ray analyses confirmed the 2D NMR data and allowed us to define the absolute configuration of the exocyclic substituent at the nitrogen atom to be *R* (Fig. 1). The tetrahydroisoquinoline unit is substituted by methyl group in position 3, a phenyl substituent in position 4, both in axial conformation, and a (1-phenylethyl) group at the nitrogen atom (Fig.1). The six-heteromembered ring adopts a screw-boat conformation (rather than a half-chair one as previously described), as indicated by puckering analysis [Q = 0.474 (6) Å, θ = 113.8 (7)°, φ = 93.9 (8) °] (Cremer & Pople, 1975). The two phenyl rings in position C4 and C18 are almost facing each other with a dihedral angle of 19.3 (4)° but with a rather long centroid-centroid distance of 4.129 (3)Å. In the crystal of the iminium salt, significant contacts between cationic species are uniquely mediated by BF4- anions, each of them being surrounded by five cations (Fig. 2). The tetrafluoridoborate anions are involved in intensive thermal motion, thus some B–F bond lengths and angles [range from 1.281 (10) to 1.327 (10) Å and from 98.9 (9) to 116.6 (8)°, respectively] deviate significantly from their standard values.

### 2. Experimental

Title compound was prepared by reaction of (3R,4S)-3-methyl-4-phenyl-3,4-dihydroisoquinoleine (1.990 g, 9 mmol) and rac-(1-bromoethyl) benzene (6.2 ml, 45 mmol). The mixture was heated at 318 K for 36 h. The reaction was monitored by TLC.

Two diastereoisomers were obtained in 1:1 ratio. These diastereoisomers were separated by column chromatography. Each compound was treated by 1 equiv of AgBF4 in acetonitrile. Filtration and concentration *in vacuo* afforded a white solid.

Only one diastereoisomer (the title compound) was successfully recrystallized. Crystals were grown by placing a solution of this dastereoisomer (45mg) in CH_3_COCH_3_ (0.5 ml) at the bottom of a test tube, then carefully covering it with pure hexane (50 ml).The test tube was covered and left undisturbed.

Colorless crystals of the title compound appeared after several days. [α]*_D_*^24^ = -16.1 (c 0.4; CHCl_3_), m.p. 447 K.

### 3. Refinement

All H atoms were positioned geometrically and treated as riding, with C—H = 0.93 (aromatic), 0.96 (methyl), or 0.98 Å (methine), with Uiso(H) = xUeq(C) where x = 1.5 for methyl H and 1.2 for all other H atoms.

The systematic absences permitted P4_1_2_1_2 and P4_3_2_1_2 as possible space groups, but in the absence of significant resonant scattering, it was not possible to distinguish between these enantiomeric space groups and the Friedel-equivalent reflections were merged. P4_1_2_1_2 was selected because the enantiomer has been assigned by reference to unchanging chiral centres in the synthetic procedure.

### Crystal data

**Table:**

C_24_H_24_N^+^·BF_4_^−^	*D*_x_ = 1.273 Mg m^−^^3^
*M**_r_* = 413.25	Cu *K*α radiation, λ = 1.5418 Å
Tetragonal, *P*4_1_2_1_2	Cell parameters from 25 reflections
Hall symbol: P 4abw 2nw	θ = 7.9–13.4°
*a* = 9.367 (4) Å	µ = 0.81 mm^−^^1^
*c* = 49.137 (14) Å	*T* = 293 K
*V* = 4311 (3) Å^3^	Prism, colourless
*Z* = 8	0.33 × 0.26 × 0.26 mm
*F*(000) = 1728	

### Data collection

**Table:**

Nonius CAD-4 diffractometer	*R*_int_ = 0.092
Radiation source: X-ray tube	θ_max_ = 67.0°, θ_min_ = 3.6°
Graphite monochromator	*h* = −11→10
θ/2θ scans	*k* = −3→11
10992 measured reflections	*l* = 0→58
2353 independent reflections	3 standard reflections every 60 min
1192 reflections with *I* > 2σ(*I*)	intensity decay: 1%

### Refinement

**Table:**

Refinement on *F*^2^	Primary atom site location: structure-invariant direct methods
Least-squares matrix: full	Secondary atom site location: difference Fourier map
*R*[*F*^2^ > 2σ(*F*^2^)] = 0.065	Hydrogen site location: difference Fourier map
*wR*(*F*^2^) = 0.215	H-atom parameters constrained
*S* = 1.08	*w* = 1/[σ^2^(*F*_o_^2^) + (0.1069*P*)^2^ + 0.3684*P*] where *P* = (*F*_o_^2^ + 2*F*_c_^2^)/3
2353 reflections	(Δ/σ)_max_ = 0.001
273 parameters	Δρ_max_ = 0.30 e Å^−^^3^
0 restraints	Δρ_min_ = −0.18 e Å^−^^3^

### Special details

**Table:**

Geometry. All e.s.d.'s (except the e.s.d. in the dihedral angle between two l.s. planes) are estimated using the full covariance matrix. The cell e.s.d.'s are taken into account individually in the estimation of e.s.d.'s in distances, angles and torsion angles; correlations between e.s.d.'s in cell parameters are only used when they are defined by crystal symmetry. An approximate (isotropic) treatment of cell e.s.d.'s is used for estimating e.s.d.'s involving l.s. planes.
Refinement. Refinement of *F*^2^ against ALL reflections. The weighted *R*-factor *wR* and goodness of fit *S* are based on *F*^2^, conventional *R*-factors *R* are based on *F*, with *F* set to zero for negative *F*^2^. The threshold expression of *F*^2^ > σ(*F*^2^) is used only for calculating *R*-factors(gt) *etc*. and is not relevant to the choice of reflections for refinement. *R*-factors based on *F*^2^ are statistically about twice as large as those based on *F*, and *R*- factors based on ALL data will be even larger.

### Fractional atomic coordinates and isotropic or equivalent isotropic displacement parameters (Å^2^)

**Table:**

	*x*	*y*	*z*	*U*_iso_*/*U*_eq_	
C1	0.5567 (6)	0.4341 (7)	0.05389 (13)	0.0603 (17)	
H1	0.5550	0.5320	0.0507	0.068*	
N2	0.5135 (5)	0.3893 (5)	0.07701 (9)	0.0529 (13)	
C3	0.5210 (7)	0.2316 (6)	0.08264 (11)	0.0539 (16)	
H3	0.4509	0.2086	0.0968	0.060*	
C4	0.4824 (6)	0.1472 (6)	0.05687 (10)	0.0517 (15)	
H4	0.5096	0.0477	0.0602	0.058*	
C5	0.6173 (8)	0.1095 (8)	0.01273 (13)	0.0717 (19)	
H5	0.5933	0.0131	0.0129	0.080*	
C6	0.6996 (9)	0.1639 (10)	−0.00821 (14)	0.085 (2)	
H6	0.7334	0.1031	−0.0217	0.095*	
C7	0.7322 (8)	0.3084 (10)	−0.00929 (14)	0.083 (2)	
H7	0.7864	0.3444	−0.0236	0.093*	
C8	0.6845 (8)	0.3968 (8)	0.01059 (12)	0.072 (2)	
H8	0.7032	0.4941	0.0096	0.081*	
C9	0.6083 (6)	0.3428 (7)	0.03230 (11)	0.0565 (16)	
C10	0.5708 (6)	0.1985 (7)	0.03335 (12)	0.0552 (16)	
C11	0.6686 (7)	0.1929 (8)	0.09325 (14)	0.077 (2)	
H11A	0.6783	0.0909	0.0938	0.088*	
H11B	0.6806	0.2311	0.1112	0.088*	
H11C	0.7399	0.2321	0.0814	0.088*	
C12	0.3215 (7)	0.1476 (7)	0.05072 (12)	0.0581 (17)	
C13	0.2383 (8)	0.0355 (8)	0.05968 (13)	0.074 (2)	
H13	0.2807	−0.0381	0.0695	0.083*	
C14	0.0943 (8)	0.0300 (9)	0.05445 (14)	0.085 (2)	
H14	0.0411	−0.0479	0.0604	0.095*	
C15	0.0293 (8)	0.1376 (11)	0.04074 (16)	0.090 (3)	
H15	−0.0683	0.1350	0.0373	0.100*	
C16	0.1104 (9)	0.2492 (10)	0.03213 (16)	0.100 (3)	
H16	0.0672	0.3234	0.0226	0.113*	
C17	0.2526 (8)	0.2549 (9)	0.03710 (15)	0.087 (2)	
H17	0.3046	0.3334	0.0311	0.097*	
C18	0.4575 (7)	0.4894 (7)	0.09788 (11)	0.0611 (17)	
H18	0.4812	0.5856	0.0915	0.068*	
C19	0.5320 (8)	0.4718 (8)	0.12507 (14)	0.087 (2)	
H19A	0.5069	0.5500	0.1368	0.100*	
H19B	0.6335	0.4708	0.1223	0.100*	
H19C	0.5027	0.3836	0.1333	0.100*	
C20	0.2977 (7)	0.4823 (7)	0.09907 (12)	0.0562 (16)	
C21	0.2243 (8)	0.3923 (8)	0.11650 (14)	0.079 (2)	
H21	0.2736	0.3317	0.1282	0.088*	
C22	0.0726 (9)	0.3937 (11)	0.1163 (2)	0.102 (3)	
H22	0.0220	0.3337	0.1279	0.115*	
C23	0.0022 (10)	0.4820 (12)	0.09934 (19)	0.102 (3)	
H23	−0.0971	0.4827	0.0996	0.114*	
C24	0.0700 (10)	0.5679 (12)	0.08225 (19)	0.114 (3)	
H24	0.0190	0.6258	0.0703	0.128*	
C25	0.2198 (9)	0.5703 (9)	0.08234 (15)	0.089 (2)	
H25	0.2675	0.6330	0.0708	0.100*	
B26	0.7073 (11)	0.7827 (9)	0.0634 (2)	0.078 (3)	
F27	0.5714 (5)	0.7850 (5)	0.05584 (14)	0.151 (3)	
F28	0.7744 (6)	0.9020 (6)	0.06260 (15)	0.160 (3)	
F29	0.7185 (7)	0.7172 (11)	0.08622 (15)	0.248 (5)	
F30	0.7760 (9)	0.6905 (9)	0.0478 (2)	0.237 (5)	

### Atomic displacement parameters (Å^2^)

**Table:**

	*U*^11^	*U*^22^	*U*^33^	*U*^12^	*U*^13^	*U*^23^
C1	0.051 (4)	0.063 (4)	0.066 (4)	−0.017 (3)	0.004 (3)	0.003 (3)
N2	0.053 (3)	0.054 (3)	0.052 (3)	0.002 (3)	0.007 (2)	−0.005 (2)
C3	0.056 (4)	0.054 (4)	0.051 (3)	0.004 (3)	−0.006 (3)	0.007 (3)
C4	0.050 (4)	0.051 (4)	0.054 (3)	−0.006 (3)	−0.002 (3)	0.002 (3)
C5	0.072 (5)	0.079 (5)	0.064 (4)	0.000 (4)	−0.002 (4)	−0.013 (4)
C6	0.088 (6)	0.105 (7)	0.062 (4)	0.003 (5)	0.016 (4)	−0.013 (5)
C7	0.075 (5)	0.109 (7)	0.065 (4)	−0.006 (5)	0.021 (4)	−0.001 (5)
C8	0.072 (5)	0.082 (5)	0.063 (4)	−0.013 (4)	0.008 (4)	0.004 (4)
C9	0.052 (4)	0.068 (4)	0.050 (3)	−0.008 (3)	0.010 (3)	0.001 (3)
C10	0.052 (4)	0.061 (4)	0.053 (3)	−0.006 (3)	−0.008 (3)	−0.001 (3)
C11	0.063 (5)	0.095 (6)	0.072 (4)	0.009 (4)	−0.016 (4)	−0.004 (4)
C12	0.054 (4)	0.066 (4)	0.054 (3)	−0.016 (4)	−0.010 (3)	−0.004 (3)
C13	0.074 (5)	0.082 (5)	0.066 (4)	−0.026 (4)	−0.004 (4)	0.004 (4)
C14	0.068 (5)	0.107 (7)	0.079 (5)	−0.043 (5)	0.014 (4)	−0.018 (5)
C15	0.043 (4)	0.135 (8)	0.090 (5)	−0.009 (5)	−0.001 (4)	0.002 (6)
C16	0.067 (6)	0.115 (7)	0.119 (7)	−0.003 (5)	−0.018 (5)	0.024 (6)
C17	0.053 (5)	0.100 (6)	0.107 (5)	−0.014 (4)	−0.012 (4)	0.023 (5)
C18	0.065 (4)	0.060 (4)	0.058 (3)	0.001 (3)	0.007 (3)	−0.007 (3)
C19	0.078 (5)	0.111 (7)	0.072 (4)	0.003 (5)	−0.006 (4)	−0.023 (5)
C20	0.058 (4)	0.050 (4)	0.060 (4)	−0.001 (3)	0.007 (3)	−0.004 (3)
C21	0.075 (5)	0.074 (5)	0.087 (5)	0.005 (4)	0.008 (4)	0.022 (4)
C22	0.066 (6)	0.106 (7)	0.135 (8)	−0.011 (5)	0.030 (5)	0.008 (6)
C23	0.062 (5)	0.133 (9)	0.110 (7)	0.021 (6)	0.008 (5)	−0.015 (6)
C24	0.076 (7)	0.164 (11)	0.103 (7)	0.029 (6)	−0.003 (5)	0.003 (7)
C25	0.088 (6)	0.100 (7)	0.080 (5)	0.008 (5)	0.015 (5)	0.014 (5)
B26	0.078 (7)	0.047 (5)	0.108 (7)	−0.003 (5)	−0.021 (6)	−0.003 (5)
F27	0.085 (4)	0.079 (3)	0.287 (7)	−0.014 (3)	−0.087 (4)	0.040 (4)
F28	0.098 (4)	0.106 (4)	0.277 (7)	−0.051 (3)	−0.015 (4)	−0.001 (5)
F29	0.135 (6)	0.415 (15)	0.194 (6)	−0.101 (7)	−0.062 (5)	0.166 (9)
F30	0.176 (8)	0.193 (8)	0.343 (11)	0.014 (6)	0.014 (7)	−0.147 (8)

### Geometric parameters (Å, º)

**Table:**

C1—N2	1.277 (7)	C14—C15	1.356 (10)
C1—C9	1.446 (8)	C14—H14	0.9300
C1—H1	0.9300	C15—C16	1.360 (11)
N2—C18	1.485 (7)	C15—H15	0.9300
N2—C3	1.505 (7)	C16—C17	1.356 (10)
C3—C11	1.521 (8)	C16—H16	0.9300
C3—C4	1.536 (8)	C17—H17	0.9300
C3—H3	0.9800	C18—C20	1.500 (9)
C4—C10	1.500 (8)	C18—C19	1.516 (9)
C4—C12	1.537 (8)	C18—H18	0.9800
C4—H4	0.9800	C19—H19A	0.9600
C5—C10	1.383 (9)	C19—H19B	0.9600
C5—C6	1.383 (9)	C19—H19C	0.9600
C5—H5	0.9300	C20—C25	1.374 (9)
C6—C7	1.389 (10)	C20—C21	1.385 (9)
C6—H6	0.9300	C21—C22	1.421 (11)
C7—C8	1.356 (9)	C21—H21	0.9300
C7—H7	0.9300	C22—C23	1.347 (11)
C8—C9	1.379 (8)	C22—H22	0.9300
C8—H8	0.9300	C23—C24	1.325 (12)
C9—C10	1.398 (8)	C23—H23	0.9300
C11—H11A	0.9600	C24—C25	1.403 (11)
C11—H11B	0.9600	C24—H24	0.9300
C11—H11C	0.9600	C25—H25	0.9300
C12—C17	1.369 (10)	B26—F29	1.281 (10)
C12—C13	1.380 (9)	B26—F28	1.283 (9)
C13—C14	1.374 (10)	B26—F30	1.323 (11)
C13—H13	0.9300	B26—F27	1.327 (10)
			
N2—C1—C9	124.4 (6)	C15—C14—C13	120.4 (8)
N2—C1—H1	117.8	C15—C14—H14	119.8
C9—C1—H1	117.8	C13—C14—H14	119.8
C1—N2—C18	121.3 (5)	C14—C15—C16	118.3 (7)
C1—N2—C3	118.1 (5)	C14—C15—H15	120.8
C18—N2—C3	120.6 (5)	C16—C15—H15	120.8
N2—C3—C11	109.9 (5)	C17—C16—C15	121.5 (8)
N2—C3—C4	110.0 (4)	C17—C16—H16	119.2
C11—C3—C4	112.0 (5)	C15—C16—H16	119.2
N2—C3—H3	108.3	C16—C17—C12	121.5 (8)
C11—C3—H3	108.3	C16—C17—H17	119.2
C4—C3—H3	108.3	C12—C17—H17	119.2
C10—C4—C3	109.9 (5)	N2—C18—C20	110.6 (5)
C10—C4—C12	112.9 (5)	N2—C18—C19	112.2 (5)
C3—C4—C12	113.1 (5)	C20—C18—C19	114.8 (5)
C10—C4—H4	106.9	N2—C18—H18	106.2
C3—C4—H4	106.9	C20—C18—H18	106.2
C12—C4—H4	106.9	C19—C18—H18	106.2
C10—C5—C6	119.9 (7)	C18—C19—H19A	109.5
C10—C5—H5	120.0	C18—C19—H19B	109.5
C6—C5—H5	120.0	H19A—C19—H19B	109.5
C5—C6—C7	120.7 (7)	C18—C19—H19C	109.5
C5—C6—H6	119.7	H19A—C19—H19C	109.5
C7—C6—H6	119.7	H19B—C19—H19C	109.5
C8—C7—C6	119.7 (7)	C25—C20—C21	118.2 (7)
C8—C7—H7	120.2	C25—C20—C18	118.7 (6)
C6—C7—H7	120.2	C21—C20—C18	123.1 (6)
C7—C8—C9	120.2 (7)	C20—C21—C22	119.1 (7)
C7—C8—H8	119.9	C20—C21—H21	120.4
C9—C8—H8	119.9	C22—C21—H21	120.4
C8—C9—C10	120.9 (6)	C23—C22—C21	120.0 (8)
C8—C9—C1	121.6 (6)	C23—C22—H22	120.0
C10—C9—C1	117.4 (5)	C21—C22—H22	120.0
C5—C10—C9	118.5 (6)	C24—C23—C22	122.0 (9)
C5—C10—C4	123.0 (6)	C24—C23—H23	119.0
C9—C10—C4	118.5 (5)	C22—C23—H23	119.0
C3—C11—H11A	109.5	C23—C24—C25	119.2 (9)
C3—C11—H11B	109.5	C23—C24—H24	120.4
H11A—C11—H11B	109.5	C25—C24—H24	120.4
C3—C11—H11C	109.5	C20—C25—C24	121.5 (8)
H11A—C11—H11C	109.5	C20—C25—H25	119.2
H11B—C11—H11C	109.5	C24—C25—H25	119.2
C17—C12—C13	116.6 (6)	F29—B26—F28	113.9 (9)
C17—C12—C4	124.1 (6)	F29—B26—F30	98.9 (9)
C13—C12—C4	119.3 (6)	F28—B26—F30	108.2 (9)
C14—C13—C12	121.5 (8)	F29—B26—F27	109.4 (9)
C14—C13—H13	119.2	F28—B26—F27	116.6 (8)
C12—C13—H13	119.2	F30—B26—F27	108.3 (8)
			
C9—C1—N2—C18	−178.0 (5)	C3—C4—C12—C17	83.7 (7)
C9—C1—N2—C3	2.2 (9)	C10—C4—C12—C13	139.5 (6)
C1—N2—C3—C11	85.5 (6)	C3—C4—C12—C13	−95.0 (7)
C18—N2—C3—C11	−94.3 (6)	C17—C12—C13—C14	1.9 (10)
C1—N2—C3—C4	−38.2 (7)	C4—C12—C13—C14	−179.3 (6)
C18—N2—C3—C4	141.9 (5)	C12—C13—C14—C15	−1.5 (11)
N2—C3—C4—C10	51.8 (6)	C13—C14—C15—C16	0.7 (12)
C11—C3—C4—C10	−70.7 (6)	C14—C15—C16—C17	−0.4 (13)
N2—C3—C4—C12	−75.3 (6)	C15—C16—C17—C12	0.8 (14)
C11—C3—C4—C12	162.2 (6)	C13—C12—C17—C16	−1.5 (11)
C10—C5—C6—C7	−2.2 (11)	C4—C12—C17—C16	179.7 (7)
C5—C6—C7—C8	0.9 (12)	C1—N2—C18—C20	103.3 (7)
C6—C7—C8—C9	2.3 (11)	C3—N2—C18—C20	−76.9 (7)
C7—C8—C9—C10	−4.3 (10)	C1—N2—C18—C19	−127.1 (6)
C7—C8—C9—C1	179.6 (6)	C3—N2—C18—C19	52.7 (8)
N2—C1—C9—C8	−164.3 (6)	N2—C18—C20—C25	−88.9 (7)
N2—C1—C9—C10	19.5 (9)	C19—C18—C20—C25	142.9 (7)
C6—C5—C10—C9	0.3 (10)	N2—C18—C20—C21	91.9 (7)
C6—C5—C10—C4	−179.4 (6)	C19—C18—C20—C21	−36.3 (9)
C8—C9—C10—C5	2.9 (9)	C25—C20—C21—C22	0.4 (11)
C1—C9—C10—C5	179.2 (6)	C18—C20—C21—C22	179.6 (7)
C8—C9—C10—C4	−177.4 (6)	C20—C21—C22—C23	−0.1 (13)
C1—C9—C10—C4	−1.1 (9)	C21—C22—C23—C24	0.9 (15)
C3—C4—C10—C5	146.2 (6)	C22—C23—C24—C25	−2.0 (16)
C12—C4—C10—C5	−86.6 (7)	C21—C20—C25—C24	−1.5 (12)
C3—C4—C10—C9	−33.5 (8)	C18—C20—C25—C24	179.3 (7)
C12—C4—C10—C9	93.7 (7)	C23—C24—C25—C20	2.3 (15)
C10—C4—C12—C17	−41.8 (9)		

### Hydrogen-bond geometry (Å, º)

**Table:**

*D*—H···*A*	*D*—H	H···*A*	*D*···*A*	*D*—H···*A*
C1—H1···F27	0.93	2.39	3.292 (9)	164
C1—H1···F30	0.93	2.55	3.170 (12)	125
C18—H18···F29	0.98	2.55	3.292 (10)	132
C24—H24···F30^i^	0.93	2.60	3.430 (15)	148
C4—H4···F27^ii^	0.98	2.54	3.498 (8)	165
C11—H11*A*···F28^ii^	0.96	2.50	3.268 (11)	137
C14—H14···F28^iii^	0.93	2.54	3.249 (10)	133
C7—H7···F27^iv^	0.93	2.65	3.399 (10)	138

Symmetry codes: (i) *x*−1, *y*, *z*; (ii) *x*, *y*−1, *z*; (iii) *x*−1, *y*−1, *z*; (iv) *y*, *x*, −*z*.
